# A SEER-based analysis of trends in HPV-associated oropharyngeal squamous cell carcinoma

**DOI:** 10.1186/s13027-024-00592-5

**Published:** 2024-06-28

**Authors:** Su Il Kim, Jung Woo Lee, Young-Gyu Eun, Young Chan Lee

**Affiliations:** 1 Department of Otolaryngology-Head and Neck Surgery, Kyung Hee University School of Medicine, Kyung Hee University Hospital at Gangdong, #892 Dongnamro, Gangdong-gu, Seoul, 05278 Korea; 2 Department of Oral and Maxillofacial Surgery, School of Dentistry, Kyung Hee University, Seoul, Korea; 3 Department of Otolaryngology-Head and Neck Surgery, Kyung Hee University School of Medicine, Kyung Hee University Medical Center, Seoul, Korea; 4 Department of Age Service-Tech Convergence, College of Medicine, Kyung Hee University, Seoul, Korea

**Keywords:** Oropharyngeal squamous cell carcinoma, HPV status, Epidemiology, SEER database, The autoregressive integrated moving average (ARIMA) model

## Abstract

**Background:**

The proportional trends of HPV-associated oropharyngeal squamous cell carcinoma (OPSCC) according to various factors have not been analyzed in detail in previous studies. We aimed to evaluate the trends of HPV-associated OPSCC in the United States.

**Methods:**

This retrospective cohort study included 13,081 patients with OPSCC from large population-based data using Surveillance, Epidemiology, and End Results (SEER) 2010–2017 database, 17 Registries. Patients were diagnosed with OPSCC primarily in the base of tongue (BOT), posterior pharyngeal wall (PPW), soft palate (SP), and tonsil and were tested for HPV infection status. We analyzed how the proportional trends of patients with OPSCC changed according to various demographic factors. Additionally, we forecasted and confirmed the trend of HPV (+) and (−) patients with OPSCC using the autoregressive integrated moving average (ARIMA) model.

**Results:**

The proportion of patients who performed the HPV testing increased every year, and it has exceeded 50% since 2014 (21.95% and 51.37% at 2010 and 2014, respectively). The HPV-positive rates tended to increase over past 7 years (66.37% and 79.32% at 2010 and 2016, respectively). Positivity rates of HPV were significantly higher in OPSCC located in the tonsil or BOT than in those located in PPW or SP. The ARIMA (2,1,0) and (0,1,0) models were applied to forecast HPV (+) and (−) patients with OPSCC, respectively, and the predicted data generally matched the actual data well.

**Conclusion:**

This large population-based study suggests that the proportional trends of HPV (+) patients with OPSCC has increased and will continue to increase. However, the trends of HPV (+) and (−) patients differed greatly according to various demographic factors. These results present a direction for establishing appropriate preventive measures to deal with HPV-related OPSCC in more detail.

**Supplementary Information:**

The online version contains supplementary material available at 10.1186/s13027-024-00592-5.

## Background

Head and neck squamous cell carcinoma (HNSCC) is the sixth most common cancer worldwide and includes all cancers occurring in the mucosa of the oral cavity, oropharynx, larynx, and hypopharynx [1]. Unlike the incidence rates of other HNSCCs, which have declined in recent years, the incidence of oropharyngeal squamous cell carcinoma (OPSCC) has shown a significant increase worldwide [2]. This phenomenon is attributed to greater prevalence of infections caused by human papillomavirus (HPV), which is essential for the etiology of cervical cancer. Transmission of HPV, which occurs primarily through sexual contact, can lead to oropharyngeal HPV infection [3]. Unlike HPV positive (HPV (+)) OPSCC, HPV negative (HPV (−)) OPSCC is mainly associated with tobacco and alcohol consumption, and its incidence has reportedly decreased in recent years [4].

Interestingly, HPV (+) status in patients with OPSCC is associated with a better prognosis than an HPV (−) status [5]. The American Joint Committee on Cancer (AJCC) Staging Manual, 8th Edition, effective Jan 2018, defined HPV (+) and HPV (−) OPSCCs as separate entities, considering molecular profiles, tumor characteristics, and especially outcomes [6]. Various epidemiologic factors, such as primary sites, age, sex, race, and birth year, can also uniquely contribute to the differing characteristics of patients with HPV-associated OPSCC. However, the proportional trends of HPV-associated OPSCC according to various factors have not been analyzed in detail in previous studies.

In this study, we aimed to examine the trends of HPV-associated OPSCC using the Surveillance, Epidemiology, and End Results (SEER) database. We hypothesized that the number of HPV (+) and (−) patients with OPSCC could be predicted by examining the trends of each patient over the past few years. We also hypothesized that comparison between each group classified according to various demographic factors would help establish preventive measures.

## Methods

### Patient cohort

Patient clinical data were obtained from the SEER program of the National Cancer Institute in the United States (https://seer.cancer.gov/; approval number: 20922-Nov 2019). The inclusion criteria were as follows: (1) diagnosis of oropharyngeal cancer; (2) tumor site in the base of the tongue (BOT), posterior pharyngeal wall (PPW), soft palate (SP), or tonsil; (3) histologic behavior of squamous cell carcinoma; and (4) testing for HPV status between 2010 and 2017.

Primary sites (BOT, PPW, SP, and tonsil) of OPSCC were sub-categorized as follows: (1) BOT: “not otherwise specified (NOS)” and “lingual tonsil”; (2) PPW: “lateral wall of PPW,” “overlapping lesion of PPW,” “posterior wall of PPW,” “vallecula,” and “NOS”; (3) SP: “uvula” and “NOS”; and (4) tonsil “overlapping lesion of tonsil,” “tonsillar fossa,” “tonsillar pillar,” “tonsil,” and “NOS.” The age of patients was categorized as < 40, 40–44, 45–49, 50–54, 55–59, 60–64, 65–69, 70–74, 75–79, and ≥ 80 years”. The diagnosis year of OPSCC was classified as 2010, 2011, 2012, 2013, 2014, 2015, or 2016. The race of the patients was classified as American Indian/Alaska native, Asian or Pacific Islander, Black, White, and unknown. Tumor grade, sex, and TNM staging based on the American Joint Committee on Cancer (AJCC) Staging Manual, 8th edition, were also recorded.

### The trends of HPV-associated OPSCC according to various factors between 2010 and 2017

We analyzed how the HPV positivity rates and the proportional trends of OPSCC patients changed according to various factors from 2010 to 2017. First, HPV positivity was compared in each group classified according to the primary tumor subsite and further subdivisions. Next, the proportional trends of HPV (+) and HPV (−) patients with OPSCC were analyzed according to age, sex, and race from 2010 to 2017.

### The autoregressive integrated moving average (ARIMA) model

Based on the annual changes in HPV (+) and (−) patients, we examined whether changes in the proportional trends of patients can be appropriately predicted in the future. Thus, we forecasted and confirmed the number of HPV (+) and HPV (−) patients with OPSCC from 2015 to 2017 based on data from 2010 to 2015 using the ARIMA model. ARIMA parameters p, d, and q were used in this study. The parameters p, d, and q refer to the order of the autoregressive, integrated, and moving average parts of the model, respectively [7].

Appropriate p, d, and q values were selected using the auto.arima function in R version 4.2.0. Through the auto.arima function, the optimal model that showed either the minimum Akaike information criterion (AIC), corrected Akaike information criterion (AICc), or Bayesian information criterion (BIC) was chosen [8]. The Box-Ljung test was implemented to validate that the selected model was statistically suitable (appropriate if *p* ≥ 0.05). Thereafter, the predicted results for 2 years, expressed as 95%-lower confidence limits (95%-LCL) and 95%-upper confidence limits (95%-UCL), were compared with the actual number.

### Statistical analysis

HPV positivity rates between each group classified according to several factors were compared using the chi-square test. Bar and pie graphs were used to visualize HPV positivity between each group classified according to several factors, including primary tumor sites, age, sex, and diagnosis year. A line graph was used to visualize changes in the trends of HPV (+) and HPV (−) patients from 2010 to 2017. The ARIMA model was used for 2-year time-series prediction with 95% confidence interval (CI) in HPV (+) and (−) patients with OPSCC [9]. The R software package (http://www.r-project.org) was used for all statistical analyses. Tidyverse packages including ggplot2, TTR, and forecast packages were used in this study.

## Results

### The proportional trends of OPSCC according to various demographic factors

Using SEER Research Data (17 Registries; Nov 2022 Sub), it was confirmed that the overall number of patients with OPSCC increased except for the subsite SP from 2000 to 2020 (Fig. S1). However, we analyzed the proportional trends of patients with OPSCC from 2010 to 2017, because only the data during this period presented whether or not testing for HPV status was performed. During this period, 13,081 out of a total of 29,406 patients with OPSCC were tested for HPV status. In detail, the proportion of patients who performed the HPV testing was 50.02% (6706 out of 13,407 patients) for located in the tonsil, but 23.17% (303 out of 1308 patients) for located in SP (Fig. S2a, b). The proportion of patients who performed the HPV testing increased every year, and it has exceeded 50% since 2014 (21.95% and 51.37% at 2010 and 2014, respectively; Fig. S2c). The HPV-positive rates tended to increase from 2010 to 2017 (66.37% and 79.32% at 2010 and 2016, respectively; Fig. S2d). Overall, the number of HPV (+), HPV (−), and HPV-unknown patients with OPSCC showed a tendency to increase, maintain, and decrease, respectively (Fig. S2e).

A total of 13,081 patients with OPSCC who tested for HPV status were enrolled and analyzed in this study. The demographic characteristics of the patients are summarized in Table 1. The mean age of the patients was 60.02 years (SD, 9.80 years; range 0–99 years), and 83.55% were male. The overall HPV positivity rate was 73.57%. The TNM staging data for 2747 patients were missing in the SEER database, and other TNM staging data were rephrased according to the 8th edition of AJCC guidelines.

**Table 1:** Demographics of patients with oropharyngeal squamous cell carcinoma (OPSCC) who tested for HPV status

Characteristics	N (total = 13,081)
*Age*	
< 40 years	183 (1.40%)
40–44	412 (3.15%)
45–49	1047 (8.00%)
50–54	2112 (16.15%)
55–59	2798 (21.39%)
60–64	2556 (19.54%)
65–69	1912 (14.62%)
70–74	1073 (8.20%)
75–79	545 (4.17%)
≥ 80 years	443 (3.39%)
*Sex*	
Male	11,067 (84.60%)
Female	2014 (15.40%)
*Primary tumor site*	
*Posterior pharyngeal wall (PPW)*	
Lateral wall of PPW	61 (0.47%)
Overlapping lesion of PPW	122 (0.93%)
Posterior wall of PPW	85 (0.65%)
Vallecula	94 (0.72%)
PPW; NOS	623 (4.76%)
*Soft palate*	
Soft palate; NOS	257 (1.96%)
Uvula	46 (0.35%)
*Base of tongue*	
Base of tongue; NOS	4987 (38.12%)
Lingual tonsil	100 (0.76%)
*Tonsil*	
Overlapping lesion of tonsil	72 (0.55%)
Tonsillar fossa	672 (5.14%)
Tonsillar pillar	336 (2.57%)
Tonsil; NOS	5625 (43.01%)
*Diagnosis year*	
2010	800 (6.12%)
2011	1231 (9.41%)
2012	1592 (12.17%)
2013	1968 (15.04%)
2014	2317 (17.71%)
2015	2430 (18.58%)
2016	2743 (20.97%)
*Race*	
American Indian/Alaska native	84 (0.64%)
Asian or Pacific islander	390 (2.98%)
Black	994 (7.60%)
White	11,524 (88.1%)
Unknown	89 (0.68%)
*Tumor grade*	
Well differentiated	401 (3.07%)
Moderately differentiated	3733 (28.54%)
Poorly differentiated	5344 (40.85%)
Undifferentiated/anaplastic	150 (1.15%)
Unknown	3453 (26.40%)
*T stage (American Joint Committee on Cancer (AJCC) 8th edition)*	
T0	56 (0.43%)
TX	1089 (8.33%)
T1	2479 (18.95%)
T2	3553 (27.16%)
T3	1665 (12.73%)
T4	914 (6.99%)
T4a	416 (3.18%)
T4b	162 (1.24%)
Unknown	2747 (21.00%)
*N stage (AJCC 8th edition)*	
N0	1546 (11.82%)
NX	99 (0.76%)
N1	5588 (42.72%)
N2	1100 (8.41%)
N2a	270 (2.06%)
N2b	791 (6.05%)
N2c	436 (3.33%)
N3	504 (3.85%)
Unknown	2747 (21.00%)
*M stage (AJCC 8th edition)*	
M0	9978 (76.28%)
M1	356 (2.72%)
Unknown	2747 (21.00%)
*Clinical stage (AJCC 8th edition)*	
Stage I	4819 (36.84%)
Stage II	1748 (13.36%)
Stage III	1702 (13.01%)
Stage IV	200 (1.53%)
Stage IVA	1450 (11.08%)
Stage IVB	260 (1.99%)
Stage IVC	155 (1.18%)
Unknown	2747 (21.00%)
*HPV status*	
Positive	9624 (73.57%)
Negative	3457 (26.43%)

NOS, not otherwise specified

**Fig. 1:**
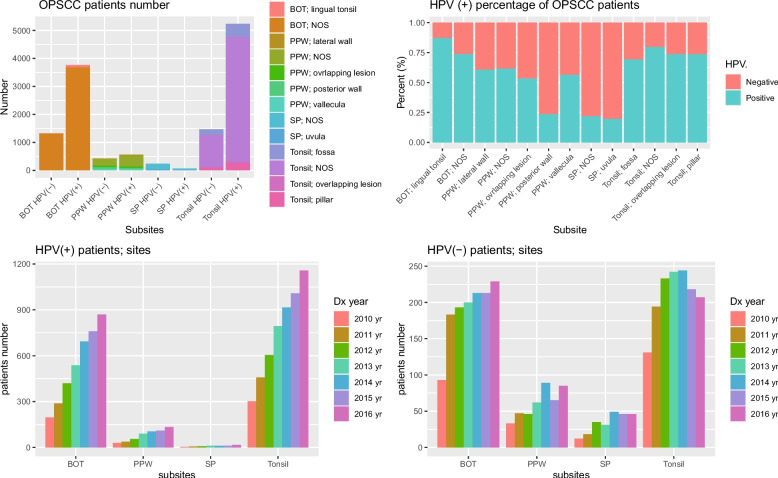
The trends in HPV-positive and HPV-negative patients with OPSCC according to the primary sites. **a** The number of HPV-positive and HPV-negative patients with OPSCC according to the sub-sites of each primary site. **b** The percentage of HPV positivity in patients with OPSCC according to the sub-sites. **c**, **d** The trends of HPV-positive and HPV-negative patients with OPSCC according to the primary sites. OPSCC, oropharyngeal squamous cell carcinoma; BOT, base of tongue; PPW, posterior pharyngeal wall; SP, soft palate

Positivity rates of HPV were significantly higher in OPSCC located in the tonsil or BOT than in those located in PPW or SP (78.09% and 73.97% in OPSCC located in the tonsil and BOT vs. 56.65% and 21.78% in OPSCC located in PPW and SP, respectively). The proportional trends in sub-sites of each primary site (BOT, PPW, SP, and tonsil) of patients with OPSCC are described in Fig. 1a. Among them, three sub-sites showed HPV-positive rates less than 25%; the HPV positivity rates for SP; uvula, SP; NOS, and PPW; posterior wall were 19.57%, 22.18%, and 23.53%, respectively (Fig. 1b). The proportional trends of HPV (+) and HPV (−) patients with OPSCC according to each primary site over the past 7 years are shown in Fig. 1c–d and Fig. S3. The number of HPV (+) patients with OPSCC generally increased over the past seven years in all age groups for each site (Fig. S3a, c, e, g). However, the number of HPV (−) patients increased until 2014 in all age groups for each site but neither changed nor decreased since then (Fig. S3b, d, g, h).

**Fig. 2:**
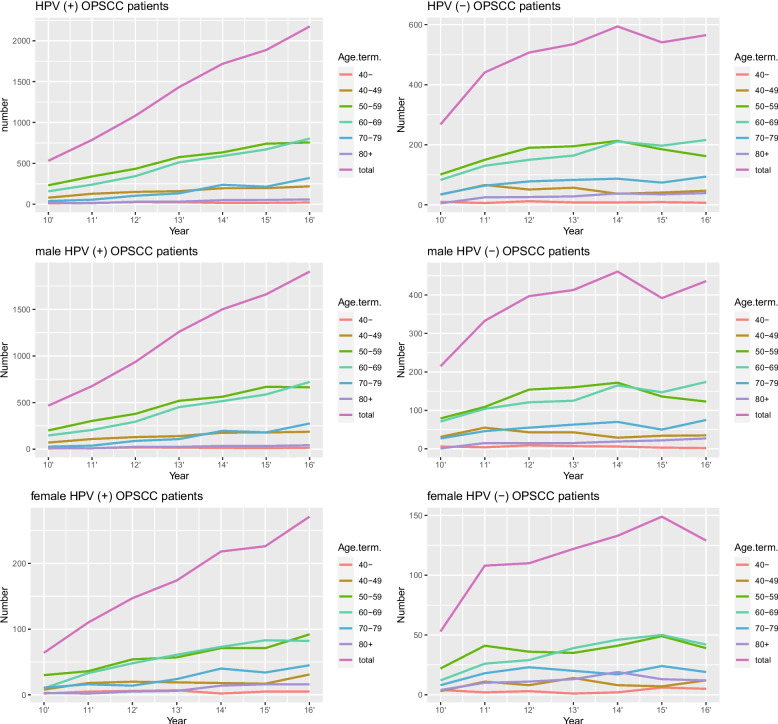
The trends in HPV-positive and HPV-negative patients with OPSCC according to the age and sex. **a**, **b** The trends of HPV-positive and HPV-negative patients with OPSCC to age group. **c**, **d** The trends of HPV-positive and HPV-negative male patients with OPSCC according to age groups. **e**, **f** The trends of HPV-positive and HPV-negative female patients with OPSCC according to age groups. OPSCC, oropharyngeal squamous cell carcinoma

Most patients with OPSCC were in the age group 55–59 years, followed by 60–64 years and 50–54 years (21.39%, 19.54%, and 16.15%, respectively; Fig. S4a). However, the HPV-positive rates were highest in the age group 45–49 years (77.27%) and showed an approximately decreasing tendency with increasing age (Fig. S4b). We analyzed the proportional trends of HPV ( +) and HPV (−) patients with OPSCC according to sex and age group over the past 7 years (Fig. 2). In all age groups, the number of male and female HPV (+) patients with OPSCC increased steadily (Fig. 2a, c, e). The increasing tendency in the number of male patients, which was rapid and disproportionate to that observed in female patients, may be attributed to the fact that there were more male patients than female patients in the study sample (11,067 vs. 2014; male vs. female patients from 2010 to 2017). The rate of increase was also greater in the age groups with the highest actual number of patients (50–59 years and 60–69 years). The trends of HPV (−) patients with OPSCC were different from that of HPV (+) patients (Fig. 2b). The number of HPV (−) patients increased until 2014 in all age and sex groups, but did not show clear increasing tendency since then (Fig. 2b, d, f). Specifically, the number of male HPV (−) patients with OPSCC has decreased between 2014 and 2015, whereas the number of female HPV (−) patients has decreased since 2015 (Fig. 2d, f).

The most predominant ethnicity was Caucasian (non-hispanic White), followed by African American (Black), and Asian and others (88.10%, 7.60%, and 4.30%, respectively). The HPV-positive rates were 75.60%, 53.40%, and 67.70% in Caucasians, African Americans, and Asian and other, respectively (Fig. S5).

### Prediction model identification and forecast using ARIMA

**Fig. 3:**
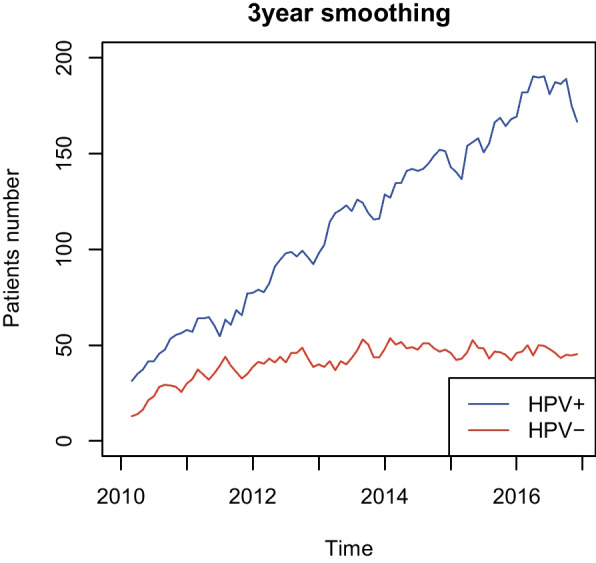
The number of monthly HPV-positive and HPV-negative patients with OPSCC from 2010 to 2017. OPSCC, oropharyngeal squamous cell carcinoma

The number of HPV ( +) patients with OPSCC have approximately increased, but the number of HPV (−) patients has not changed much from 2010 to 2017 years (32 and 162 HPV (+) patients with OPSCC in Jan 2010 and Dec 2016 vs. 16 and 43 HPV (−) patients with OPSCC in Jan 2010 and Dec 2016; Fig. 3).

We hypothesized that the proportional trends of HPV (+) and HPV (−) patients could be predicted based on the pattern of the number of patients in the past. The time-series data were divided into a training dataset (Jan 2010 to Dec 2014) and a test dataset (Jan 2015 to Dec 2016). There was no obvious periodicity or seasonality between 2010 and 2015 in HPV (+) and HPV (−) patients.

**Fig. 4:**
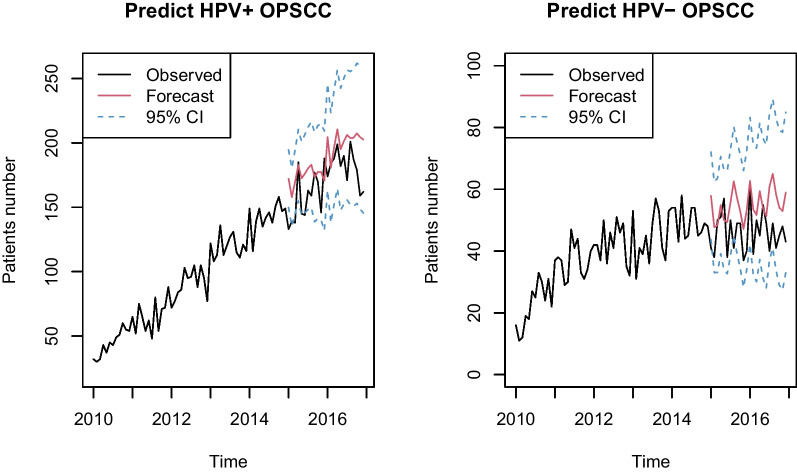
Forecasted and observed trends of HPV-positive and HPV-negative patients with OPSCC. **a** The prediction of HPV-positive patients with OPSCC from Jan 2015 to Dec 2016 using ARIMA (2,1,0). **b** The prediction of HPV-negative patients with OPSCC from Jan 2015 to Dec 2016 using ARIMA (0,1,1). OPSCC, oropharyngeal squamous cell carcinoma; ARIMA, autoregressive integrated moving average; CI, confidence interval

The suitable ARIMA model constructed using the auto.arima function in HPV (+) OPSCC was ARIMA (2,1,0) (AIC = 450.55, AICc = 451.29, BIC = 458.86). The ARIMA (2,1,0) model was applied to forecast HPV (+) patients with OPSCC, and its predicted data and 95% CI from Jan 2015 to Dec 2016 are shown in Fig. 4a. The predicted data generally matched the actual data well (all data matched to the predicted data for each month with 95% CI except in Jan 2015, Mar 2015, Apr 2015, and Jun 2015; Table S1). Figure S6 shows that the ACF of the residual sequence fell within the random CI. The Box-Ljung test showed that its residual was white noise with *p* = 0.3206 (x^2^ = 0.98663, *df* = 1), indicating that the fitted data series were random, stationary, and zero-related.

In contrast, the most suitable ARIMA model for HPV (−) OPSCC was ARIMA (0,1,1) (AIC = 402.99, AICc = 403.43, BIC = 409.23). The ARIMA (0,1,1) model was applied to forecast HPV (−) patients with OPSCC, and its predicted data and 95% CI from Jan 2015 to Dec 2016 are shown in Fig. 4b. The predicted data generally matched the actual data well (all data matched to the predicted data for each month with 95% CI except in Jan 2015 and Aug 2015; Table S2). Figure S7 shows that the ACF of the residual sequence fell within the random CI. The Box-Ljung test showed that its residual was white noise with *p* = 0.5245 (x^2^ = 0.40504, *df* = 1), indicating that the fitted data series were random, stationary, and zero-related.

## Discussion

In this large population-based proportional study using the SEER database, we found that HPV-positive rates were higher in male, Caucasian patients with tonsillar OSCC and age between 45 and 49 years. The number of HPV (+) and (−) patients with OPSCC did not differ significantly in 2010. However, increasing proportional trends of patients with OPSCC were only observed in not HPV (−) group but HPV (+) group since then. Analyzing past trends of HPV (+) and (−) patients with OPSCC, we could predict the trends of patients with OPSCC in the future.

In a previous large cohort study, tonsillar OPSCC showed a more favorable prognosis than non-tonsillar OPSCC in HPV (+) patients [10]. One systemic review found that lymphoepithelial sites of the oropharynx, such as the tonsil and tongue base, showed significantly higher HPV prevalence than non-lymphoepithelial sites of the oropharynx [11]. Our study showed that HPV positivity rates were significantly higher in the tonsillar area than in the oropharyngeal area in OPSCC. HPV-positive rates were the highest in the tonsillar and NOS areas in patients with OPSCC. We believe that further subdivision and investigation of the primary tumor location would help better predict the prognosis of patients with OPSCC.

HPV is a well-known cause of cervical, oropharyngeal, vulvar, vaginal, penile, and anal cancer [12]. The availability of prophylactic HPV vaccines has provided powerful tools for primary prevention of cervical cancers since the HPV vaccine was approved by the US Food and Drug Administration in 2006 for women and 2009 for men [13, 14]. Cervical cancers are preventable with HPV vaccine uptake and systemized screening, but other HPV-associated cancers including OPSCC do not have any screening guidelines. From 2001 to 2017, women showed the decreasing incidence of cervical cancer and stable incidence of OPSCC. However, men showed significant increase in incidence of OPSCC [14]. This may be because of significant differences by sex and anatomic sites as well as HPV vaccine policy [15]. Men seem to remain at high risk of HPV infection regardless of age. Additionally, the second peak in high-risk oropharyngeal HPV infection prevalence may occur at any age at men. It is necessary to observe the proportional trends of OPSCC for decades, because the HPV vaccine for men has been introduce since 2009, and the period is still too short for its effect to appear.

Data from the SEER database between 1973 and 2004 showed that the proportion of potentially HPV-related oral squamous cell carcinoma (OSCC) increased steadily, particularly among white men and at younger ages [16]. Potentially HPV-unrelated OSCC declined in general between 1973 and 2004. However, these previous data do not contain information about HPV status; thus, tumors arising from the oropharynx, including the tonsil and tongue base, were defined as HPV-related OSCC. A recent large population-based epidemiologic study using the SEER database between 2013 and 2014 showed that most HPV (+) patients with OPSCC were white males younger than 65 years [17]. In the present study, we specifically selected patients with OPSCC who had tested for HPV status in the SEER database between 2010 and 2017. We also found that the HPV-positive rates were higher in middle-aged white males. However, patients older than 70 years or of African American descent showed high HPV-positive rates (> 50%). A possible explanation for this finding could be that most OPSCCs in this study were tonsillar and that the HPV-positive rates in patients steadily increased every year. A large hospital-based national cohort study confirmed that the prevalence of HPV is increasing among male and female patients from White, Black, and Hispanic ethnicities [18].

The ARIMA model has been widely used in time-series analysis of various cancers [19, 20]. We also used the ARIMA model to predict the trends of HPV (+) and (−) OPSCC. To obtain appropriate ARIMA models for HPV (+) and (−) OPSCC, we first decomposed a time series into trend, seasonal, cyclical, and irregular components [9]. In our study, a seasonal tendency was not observed in both HPV (+) and (−) OPSCC; thus, the multiplicative seasonal ARIMA model was not applied [7]. The optimal ARIMA (p, d, q) model was confirmed using the auto.arima function of R 4.2.0 software. Through this function, we found optimal ARIMA (2,1,0) and (0,1,1) models for HPV (+) and HPV (−) patients with OPSCC, respectively. To check the accuracy of each model in the prediction of HPV (+) and (−) patients with OPSCC, 95% CIs were described. Some outbreaks were observed during the prediction of HPV (+) and (−) patients with OPSCC in 2015, but the forecast of HPV (+) and (−) patients matched the actual data in 2016.

Our study has the following limitations. First, we conducted a retrospective analysis of data from the SEER database, noting the absence of smoking and alcohol history, as well as molecular characterization. A systematic review and meta-analysis indicated a negative interaction between smoking and alcohol in the development of HPV (+) OPSCC [21]. In a previous study involving 157 OPSCC patients, gene alterations associated with oxidative stress were observed more frequently in HPV-positive cases, whereas those related to the p53 signaling and cell cycle control pathways were more prevalent in HPV-negative cases [22]. Furthermore, it revealed significant disparities in immune characteristics, including B cells, CD8 + T cells, fibroblasts, and M2 macrophages, among HPV-positive cancers across different anatomical sites, such as the oropharynx, non-oropharynx, and cervix [23]. Further investigation into these demographic and molecular factors is imperative for a deeper understanding of proportional trends in HPV (+) OPSCC.

Second, this study only included patients with OPSCC diagnosed between 2010 and 2017. As this study explored HPV positivity in OPSCC cases, which are already known to be related with HPV infections, we only utilized data from 2010 to 2017 because most SEER databases from the 1990s and the 2000s contain no information about HPV status. The proportion of patients who performed the HPV testing steadily increased every year. From 2010 to 2014, the HPV testing rates increased significantly (21.95% to 51.37%), thus it is thought that the number of both HPV (+) and (−) patients with OPSCC increased. From 2014 to 2017, the HPV testing rates increase to a relatively small extent (51.37% to 58.14%), and at this time, the number of HPV (−) patients with OPSCC remained unchanged. However, the number of HPV (+) patients with OPSCC continued to increase regardless of similar testing rates between this period. These phenomena suggest proportional trends in HPV (+) and (−) patients with OPSCC.

Additionally, the coronavirus disease 2019 (COVID-19) pandemic, first diagnosed in December 2019, has posed a significant threat to global public health [24]. A single-institution retrospective study indicated a delay in the diagnosis of HPV (+) OPSCC due to the COVID-19 pandemic, factors including restricted access to medical centers and unavailability of clinic appointments [25]. However, the SEER databases do not include information on the HPV status of OPSCC patients since 2017. Consequently, the impact of the COVID-19 pandemic could not be analyzed using SEER databases. Further studies utilizing a large population database that includes HPV status information up to recent periods are necessary to comprehensively analyze the impact of the COVID-19 pandemic on the proportional trends in HPV (+) OPSCC.

Third, information on HPV status is site-specifically disproportionate as HPV tests are often conducted only in areas where HPV is suspected to be positive. This is particularly relevant for tonsillar OPSCCs. In this study, the HPV testing was performed in 50.02% and 42.96% of patients with OPSCC located in the tonsil and BOT, respectively, and the positivity rates of HPV were 78.09% and 73.97% in patients. On the other hand, the HPV testing was performed in 23.17% of patients located in SP, and the positivity rates of HPV was 21.78%. Thus, the HPV testing is required to be carried out in consideration of the tumor subsites and other proportional factors of patients with OPSCC.

Nevertheless, to the best of our knowledge, this is the first study to predict future HPV trends by observing past proportional trends of OPSCC patients using a large population database. Examining the changes in HPV status in OPSCC raises the importance of preventive approaches. Appropriate screening strategies based on various factors are important to prevent the occurrence of OPSCC. Early cancer detection with HPV testing, commercialization of vaccination, and public awareness of causative factors other than HPV status are key factors in preventing OPSCC. Further studies, including more detailed epidemiological data, will help identify other factors affecting HPV status in OPSCC.

## Conclusions

In conclusion, we confirmed various proportional trends of HPV (+) and HPV (−) patients with OPSCC according to the subsite, age, sex, and ethnicity using large population-based database. In particular, the trends of HPV-related OPSCC could also be predicted by examining the trends of HPV-related OPSCC patients over the past few years. It is necessary to establish appropriate preventive measures to deal with HPV-related OPSCC more effectively according to various epidemiological factors.

### Supplementary Information

Supplementary Material 1: Table 1. The predicted and actual values of the ARIMA (2,1,0) model in HPV-positive patients with OPSCC. Supplementary Table 2. The predicted and actual values of the ARIMA (0,1,1) model in HPV-negative patients with OPSCC.

Supplementary Material 2. Figure 1. The trends in patients with OPSCC from 2000 to 2020 based on SEER Research Data (17 Registries; Nov 2022 Sub). (a) The overall number of patients with OPSCC. (b) The number of patients with OPSCC according to the primary sites. Supplementary Fig. 1 footnotes: OPSCC, oropharyngeal squamous cell carcinoma; BOT, base of tongue; PPW, posterior pharyngeal wall; SP, soft palate.

Supplementary Material 3: Figure 2. The trends in testing for HPV status in patients with OPSCC according to the primary subsites and diagnosis year from 2010 to 2017. (a–c) The percentage of patients with OPSCC who performed the HPV testing according to the primary sites, sub-sites, and the diagnosis year. (d) The percentage of HPV positivity in patients with OPSCC who performed the HPV testing according to the diagnosis year. (e) Overall HPV status of patients with OPSCC. OPSCC, oropharyngeal squamous cell carcinoma; BOT, base of tongue; PPW, posterior pharyngeal wall; SP, soft palate.

Supplementary Material 4: Figure 3. The trends in HPV-positive and HPV-negative patients with OPSCC according to age and primary sites. (a–b) The trends of HPV-positive and HPV-negative patients with OPSCC that originated in BOT, classified according to age group. (c–d) The trends of HPV-positive and HPV-negative patients with OPSCC that originated in PPW, classified according to age group. (e–f) The trends of HPV-positive and HPV-negative patients with OPSCC that originated in SP, classified according to age group. (g–h) The trends of HPV-positive and HPV-negative patients with OPSCC that originated in tonsils, classified according to age group. OPSCC, oropharyngeal squamous cell carcinoma; BOT, base of tongue; PPW, posterior pharyngeal wall; SP, soft palate.

Supplementary Material 5: Figure 4. HPV positive rates according to the age and sex of patients with OPSCC. (a) The number of HPV-positive and HPV-negative patients with OPSCC according to age and sex. (b) The percentage of HPV positivity in patients with OPSCC according to age and sex. OPSCC, oropharyngeal squamous cell carcinoma.

Supplementary Material 6: Figure 5. HPV positive rates according to the races of patients with OPSCC. OPSCC, oropharyngeal squamous cell carcinoma.

Supplementary Material 7: Figure 6. Validation of ARIMA (2,1,0) model in HPV-positive patients with OPSCC. OPSCC, oropharyngeal squamous cell carcinoma; ARIMA, autoregressive integrated moving average; ACF, autocorrelation function.

Supplementary Material 8: Figure 7. Validation of ARIMA (0,1,1) model in HPV-negative patients with OPSCC. OPSCC, oropharyngeal squamous cell carcinoma; ARIMA, autoregressive integrated moving average; ACF, autocorrelation function.

## Data Availability

The data that support the findings of our study are available from the corresponding author upon reasonable request and with permission of the SEER database.
