# Supplementary material for: A SEER-based analysis of trends in HPV-associated oropharyngeal squamous cell carcinoma
Source: Infect Agent Cancer. 2024 Jun 28;19:29. doi: 10.1186/s13027-024-00592-5 (PMC11214209; doi:10.1186/s13027-024-00592-5)
Supplement: Supplementary file 1 — Supplementary Material 1: Table 1. The predicted and actual values of the ARIMA (2,1,0) model in HPV-positive patients with OPSCC. Supplementary Table 2. The predicted and actual values of the ARIMA (0,1,1) model in HPV-negative patients with OPSCC. [file 13027_2024_592_MOESM1_ESM.docx]

**Supplementary Table Legends**

**Supplementary Table 1.** The predicted and actual values of the ARIMA (2,1,0) model in HPV-positive patients with OPSCC.

|  | Actual values | Forecast mean | 95% LCL | 95% UCL |
| --- | --- | --- | --- | --- |
| JAN 2015 | 133 | 172.1752 | 149.8061 | 194.5442 |
| FEB 2015* | 139 | 157.8572 | 135.2804 | 180.4341 |
| MAR 2015 | 138 | 169.7256 | 145.1353 | 194.3159 |
| APR 2015 | 185 | 182.7762 | 155.1498 | 210.4026 |
| MAY 2015* | 145 | 172.5186 | 143.9824 | 201.0547 |
| JUN 2015 | 144 | 176.0353 | 145.602 | 206.4685 |
| JUL 2015* | 163 | 180.3082 | 148.2597 | 212.3568 |
| AUG 2015* | 159 | 183.0595 | 149.7783 | 216.3408 |
| SEP 2015* | 177 | 173.5331 | 138.7424 | 208.3238 |
| OCT 2015* | 170 | 177.3878 | 141.2939 | 213.4817 |
| NOV 2015* | 146 | 177.454 | 140.1192 | 214.7888 |
| DEC 2015* | 188 | 170.693 | 132.084 | 209.302 |
| JAN 2016* | 174 | 204.4936 | 163.1436 | 245.8435 |
| FEB 2016* | 184 | 180.7548 | 138.1206 | 223.3891 |
| MAR 2016* | 188 | 196.5135 | 152.2272 | 240.7997 |
| APR 2016* | 199 | 210.5762 | 164.5388 | 256.6136 |
| MAY 2016* | 182 | 195.1288 | 147.7127 | 242.5449 |
| JUN 2016* | 190 | 201.3149 | 152.3684 | 250.2614 |
| JUL 2016* | 171 | 206.1545 | 155.7569 | 256.5521 |
| AUG 2016* | 201 | 203.7438 | 151.9898 | 255.4978 |
| SEP 2016* | 187 | 204.0375 | 150.9024 | 257.1726 |
| OCT 2016* | 179 | 207.4721 | 153.0181 | 261.9261 |
| NOV 2016* | 159 | 204.4548 | 148.7155 | 260.1941 |
| DEC 2016* | 162 | 202.6169 | 145.6077 | 259.6261 |

LCL, lower confidence limit; UCL, upper confidence limit

95% *

**Supplementary Table 2.** The predicted and actual values of the ARIMA (0,1,1) model in HPV-negative patients with OPSCC

|  | Actual values | Forecast mean | 95% LCL | 95% UCL |
| --- | --- | --- | --- | --- |
| JAN 2015 | 41 | 57.86635 | 43.69821 | 72.03448 |
| FEB 2015* | 38 | 47.7319 | 33.02573 | 62.43808 |
| MAR 2015* | 50 | 48.33658 | 33.11137 | 63.5618 |
| APR 2015* | 51 | 54.86179 | 39.13465 | 70.58893 |
| MAY 2015* | 57 | 50.07492 | 33.8614 | 66.28845 |
| JUN 2015* | 38 | 49.3647 | 32.67895 | 66.05044 |
| JUL 2015* | 50 | 55.70191 | 38.55695 | 72.84687 |
| AUG 2015 | 41 | 62.60531 | 45.01312 | 80.19751 |
| SEP 2015* | 49 | 57.19586 | 39.16752 | 75.22419 |
| OCT 2015* | 49 | 52.85095 | 34.39678 | 71.30513 |
| NOV 2015* | 37 | 47.1555 | 28.28509 | 66.0259 |
| DEC 2015* | 40 | 53.34303 | 34.06538 | 72.62067 |
| JAN 2016* | 61 | 62.70801 | 42.19078 | 83.22525 |
| FEB 2016* | 39 | 53.42355 | 32.35256 | 74.49454 |
| MAR 2016* | 50 | 51.70602 | 30.09546 | 73.31658 |
| APR 2016* | 45 | 59.39165 | 37.25467 | 81.52863 |
| MAY 2016* | 55 | 53.83207 | 31.1809 | 76.48324 |
| JUN 2016* | 49 | 51.2419 | 28.08795 | 74.39584 |
| JUL 2016* | 40 | 60.94993 | 37.3039 | 84.59595 |
| AUG 2016* | 49 | 65.02613 | 40.89806 | 89.15421 |
| SEP 2016* | 41 | 58.41626 | 33.81557 | 83.01694 |
| OCT 2016* | 45 | 54.06999 | 29.0056 | 79.13437 |
| NOV 2016* | 48 | 52.92577 | 27.40612 | 78.44543 |
| DEC 2016* | 43 | 58.96013 | 32.99318 | 84.92708 |

LCL, lower confidence limit; UCL, upper confidence limit

95% *
