# Supplementary figures and images for: A SEER-based analysis of trends in HPV-associated oropharyngeal squamous cell carcinoma
Source: Infect Agent Cancer. 2024 Jun 28;19:29. doi: 10.1186/s13027-024-00592-5 (PMC11214209; doi:10.1186/s13027-024-00592-5)

**A** OPSCC patients; sites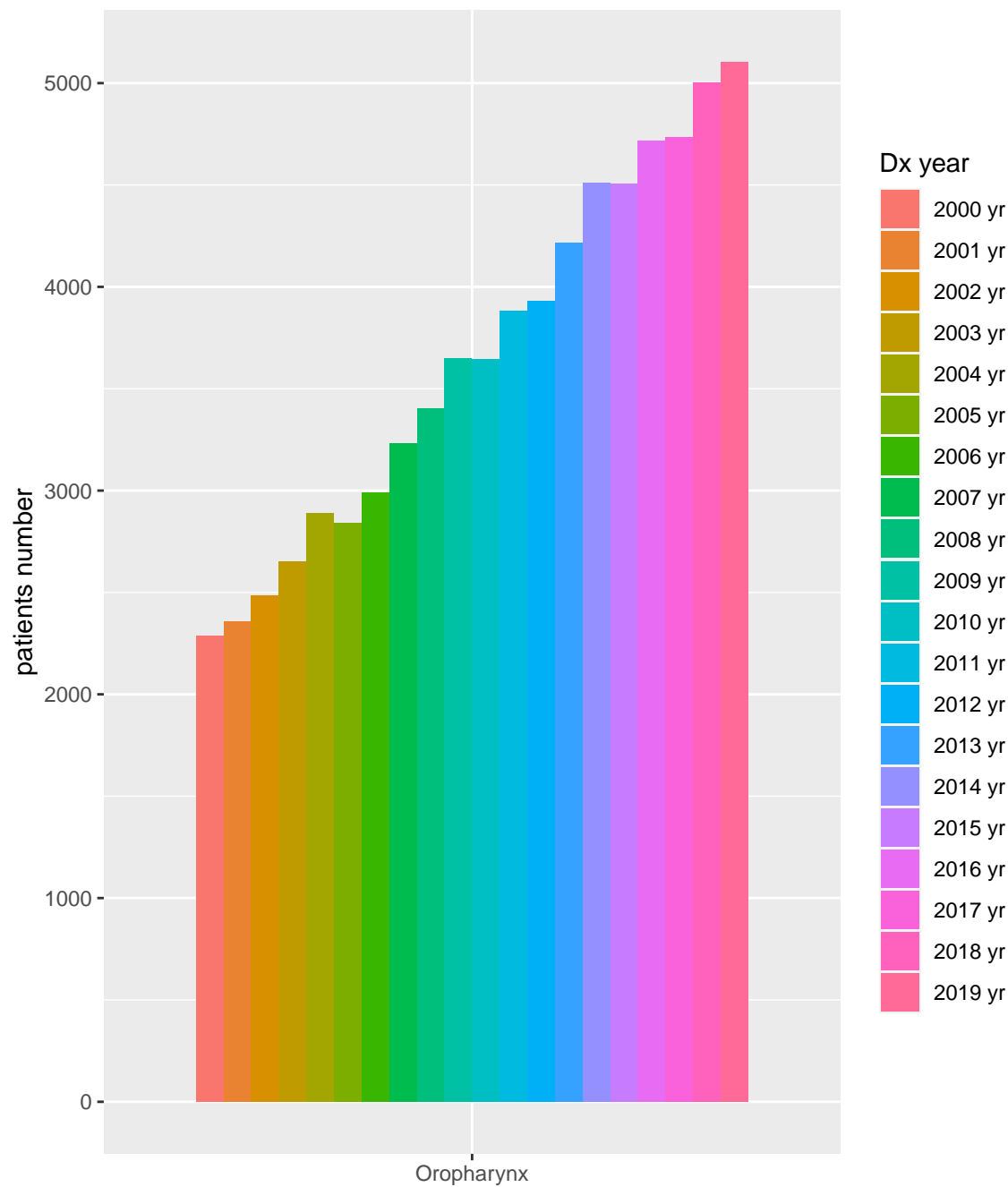**B** OPSCC patients; sites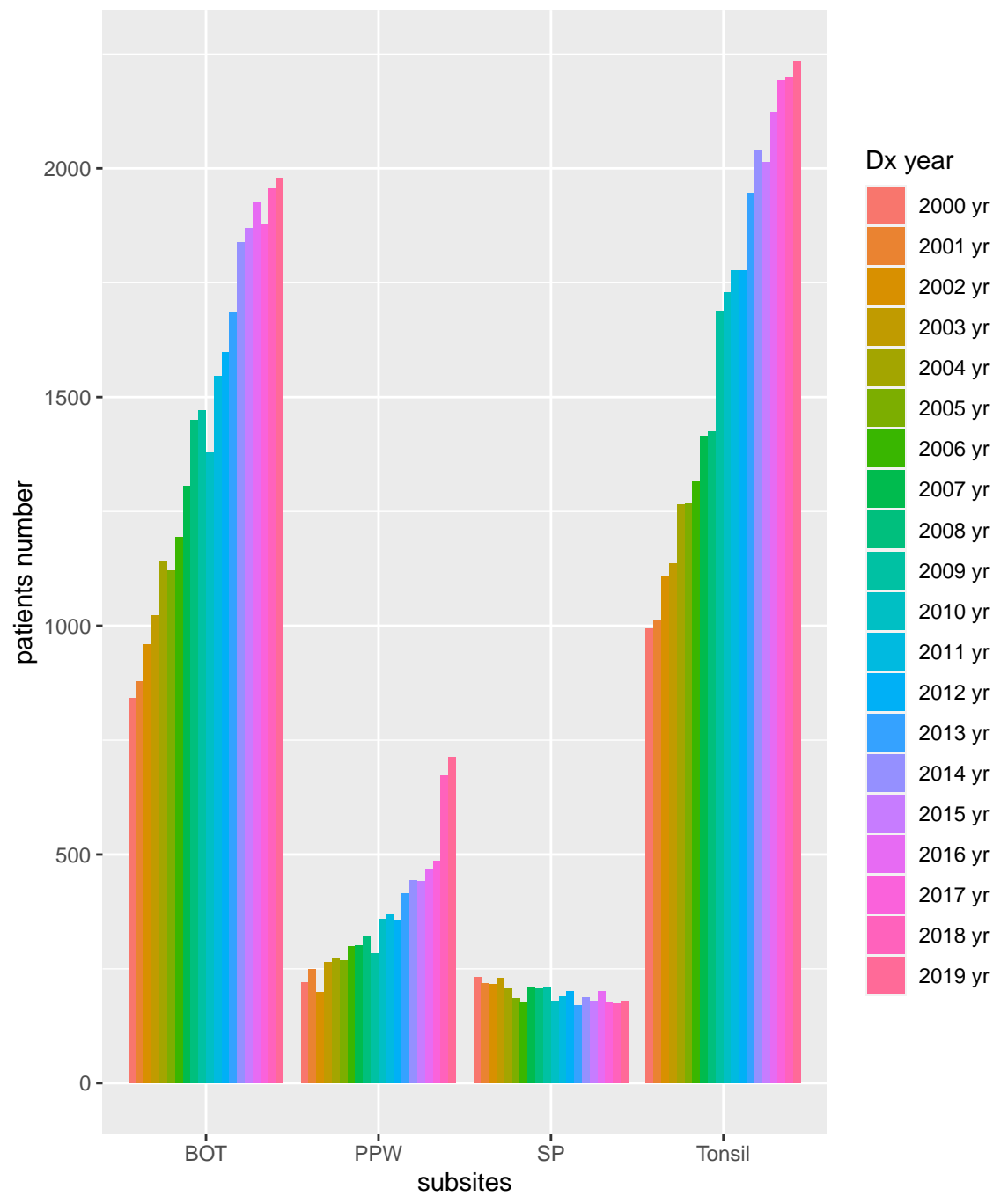

Supplement: Supplementary file 2 — Supplementary Material 2. Figure 1. The trends in patients with OPSCC from 2000 to 2020 based on SEER Research Data (17 Registries; Nov 2022 Sub). (a) The overall number of patients with OPSCC. (b) The number of patients with OPSCC according to the primary sites. Supplementary Fig. 1 footnotes: OPSCC, oropharyngeal squamous cell carcinoma; BOT, base of tongue; PPW, posterior pharyngeal wall; SP, soft palate. [file 13027_2024_592_MOESM2_ESM.pdf]

**A** Patients number; age, sex, HPV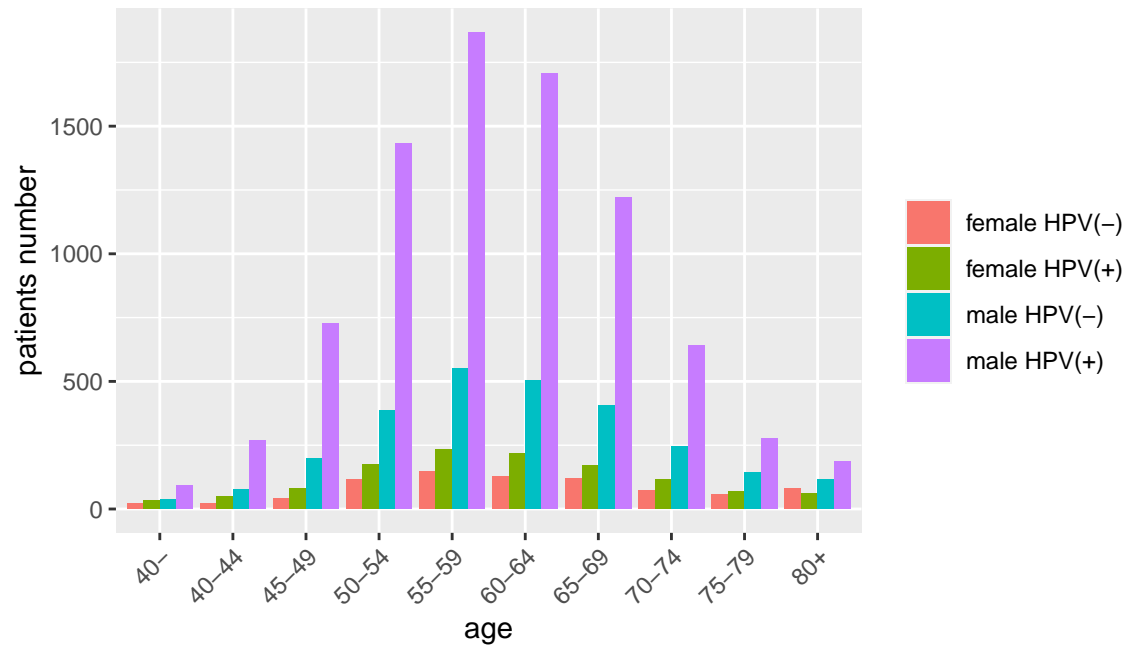**B** HPV (+) percentage; age, sex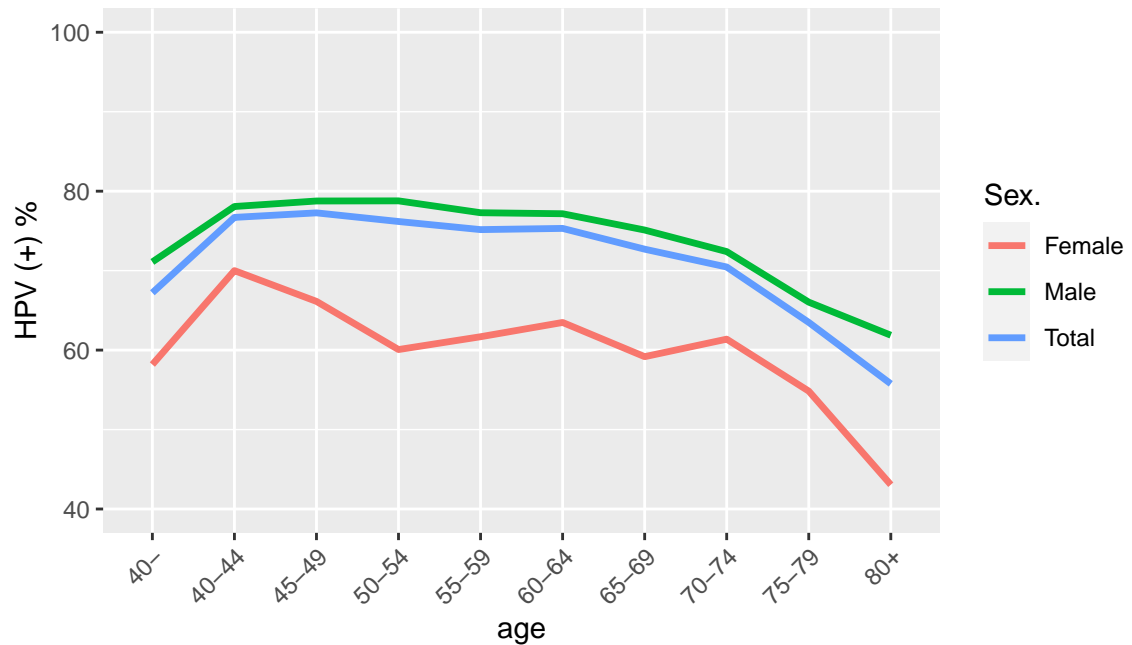

Supplement: Supplementary file 5 — Supplementary Material 5: Figure 4. HPV positive rates according to the age and sex of patients with OPSCC. (a) The number of HPV-positive and HPV-negative patients with OPSCC according to age and sex. (b) The percentage of HPV positivity in patients with OPSCC according to age and sex. OPSCC, oropharyngeal squamous cell carcinoma. [file 13027_2024_592_MOESM5_ESM.pdf]

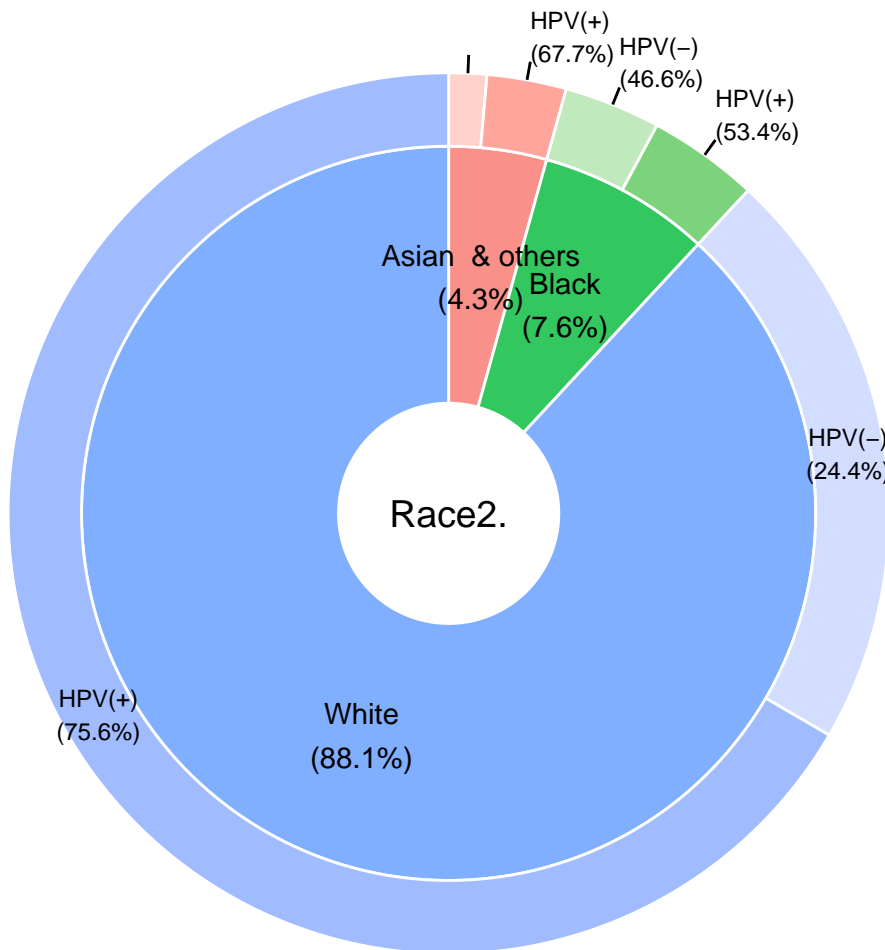

Supplement: Supplementary file 6 — Supplementary Material 6: Figure 5. HPV positive rates according to the races of patients with OPSCC. OPSCC, oropharyngeal squamous cell carcinoma. [file 13027_2024_592_MOESM6_ESM.pdf]

**Standardized Residuals**

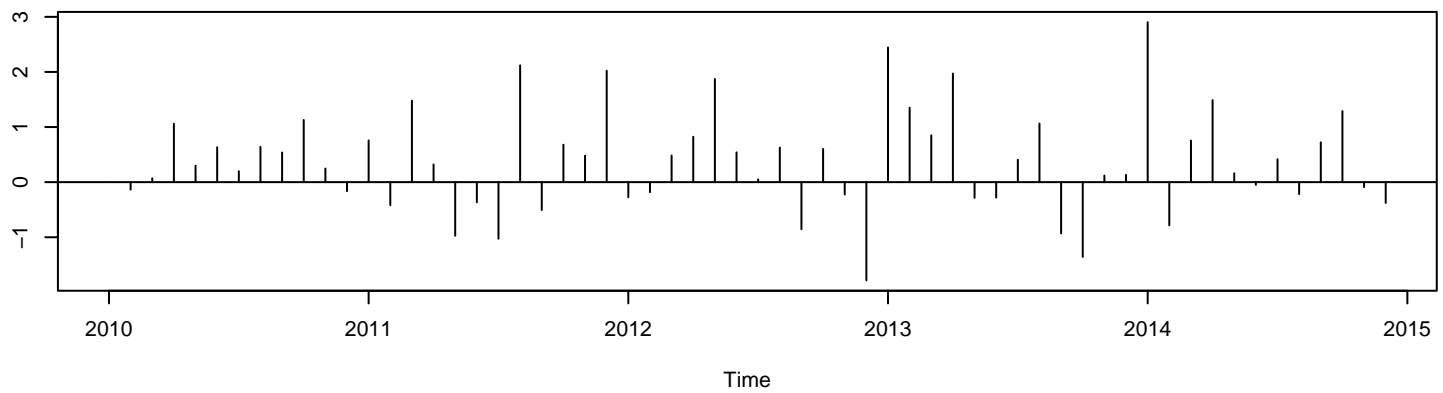

**ACF of Residuals**

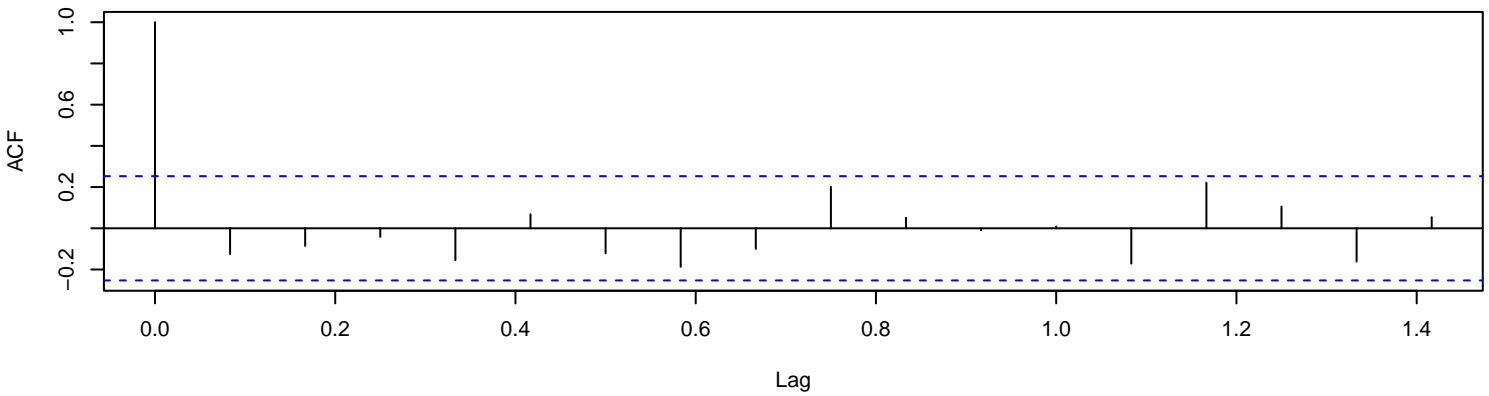

**p values for Ljung–Box statistic**

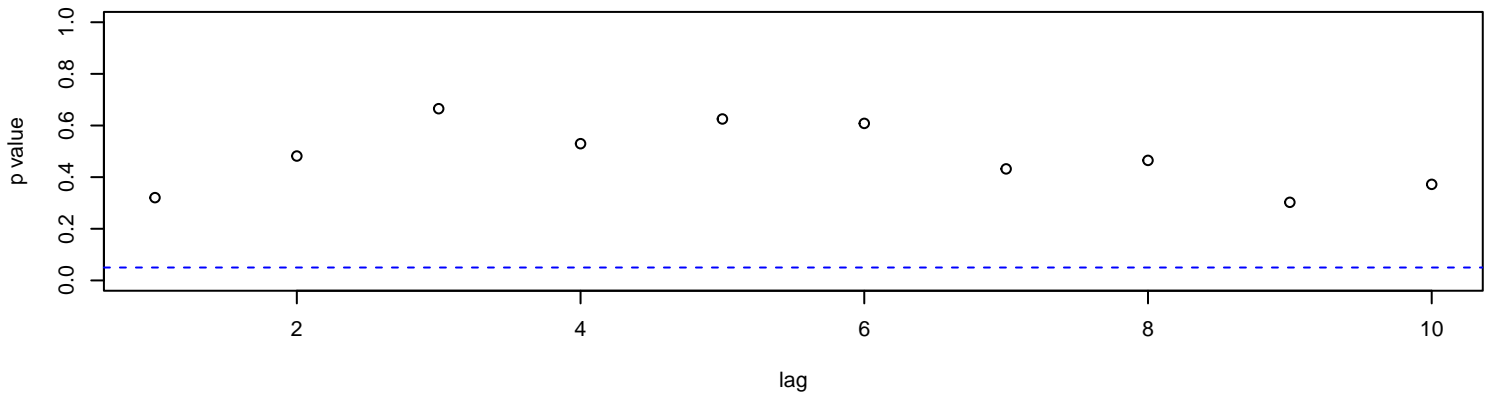

Supplement: Supplementary file 7 — Supplementary Material 7: Figure 6. Validation of ARIMA (2,1,0) model in HPV-positive patients with OPSCC. OPSCC, oropharyngeal squamous cell carcinoma; ARIMA, autoregressive integrated moving average; ACF, autocorrelation function. [file 13027_2024_592_MOESM7_ESM.pdf]

**Standardized Residuals**

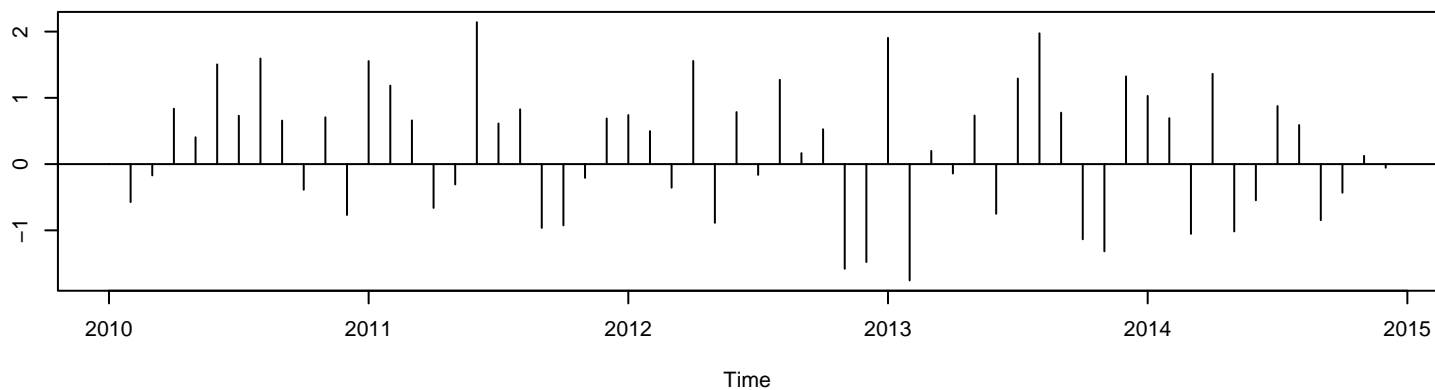

**ACF of Residuals**

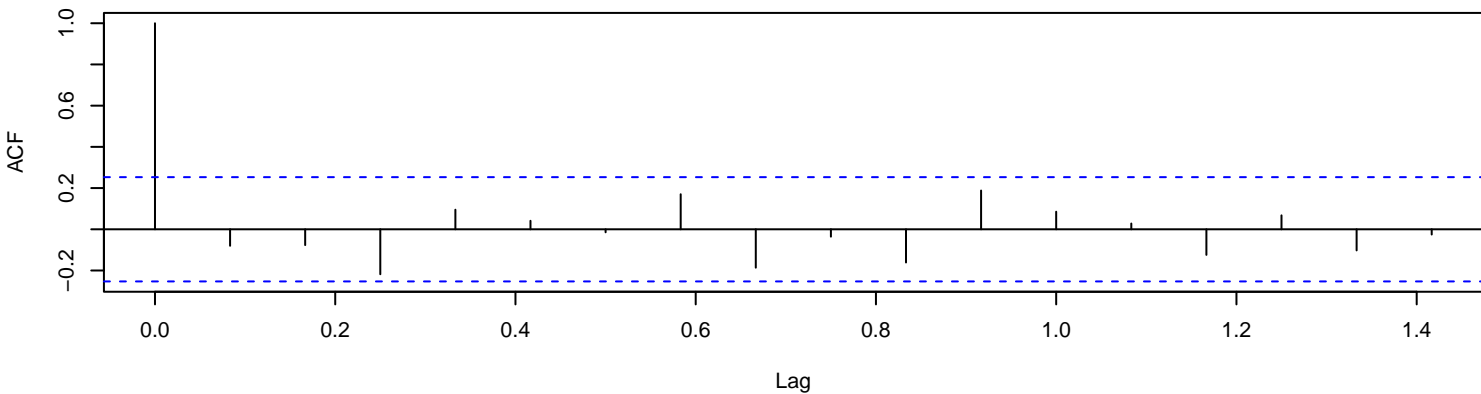

**p values for Ljung–Box statistic**

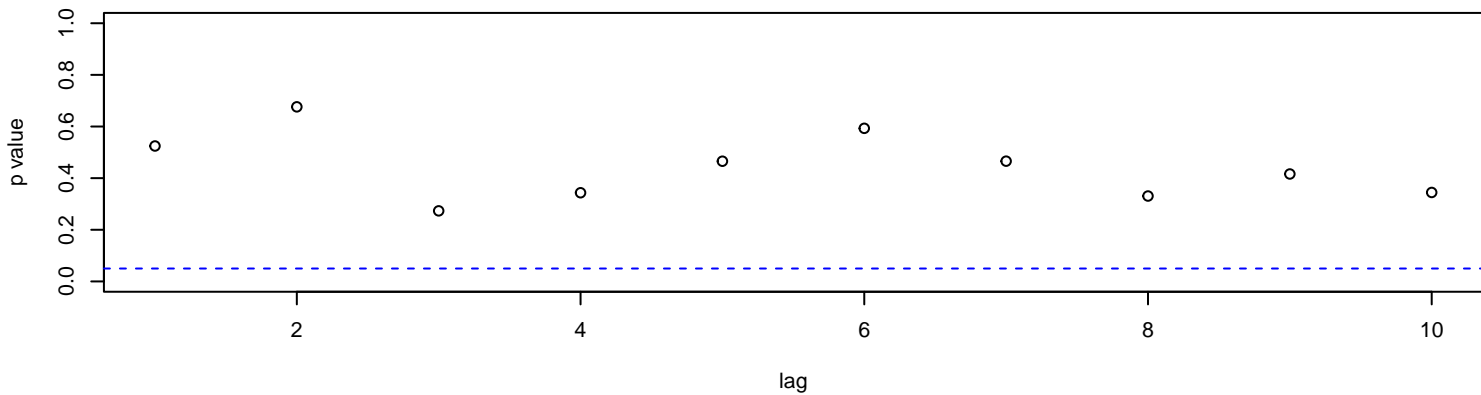

Supplement: Supplementary file 8 — Supplementary Material 8: Figure 7. Validation of ARIMA (0,1,1) model in HPV-negative patients with OPSCC. OPSCC, oropharyngeal squamous cell carcinoma; ARIMA, autoregressive integrated moving average; ACF, autocorrelation function. [file 13027_2024_592_MOESM8_ESM.pdf]
