# Supplementary material for: A SEER-based analysis of trends in HPV-associated oropharyngeal squamous cell carcinoma
Source: Infect Agent Cancer. 2024 Jun 28;19:29. doi: 10.1186/s13027-024-00592-5 (PMC11214209; doi:10.1186/s13027-024-00592-5)
Supplement: Supplementary file 3 — Supplementary Material 3: Figure 2. The trends in testing for HPV status in patients with OPSCC according to the primary subsites and diagnosis year from 2010 to 2017. (a–c) The percentage of patients with OPSCC who performed the HPV testing according to the primary sites, sub-sites, and the diagnosis year. (d) The percentage of HPV positivity in patients with OPSCC who performed the HPV testing according to the diagnosis year. (e) Overall HPV status of patients with OPSCC. OPSCC, oropharyngeal squamous cell carcinoma; BOT, base of tongue; PPW, posterior pharyngeal wall; SP, soft palate. [file 13027_2024_592_MOESM3_ESM.pdf]

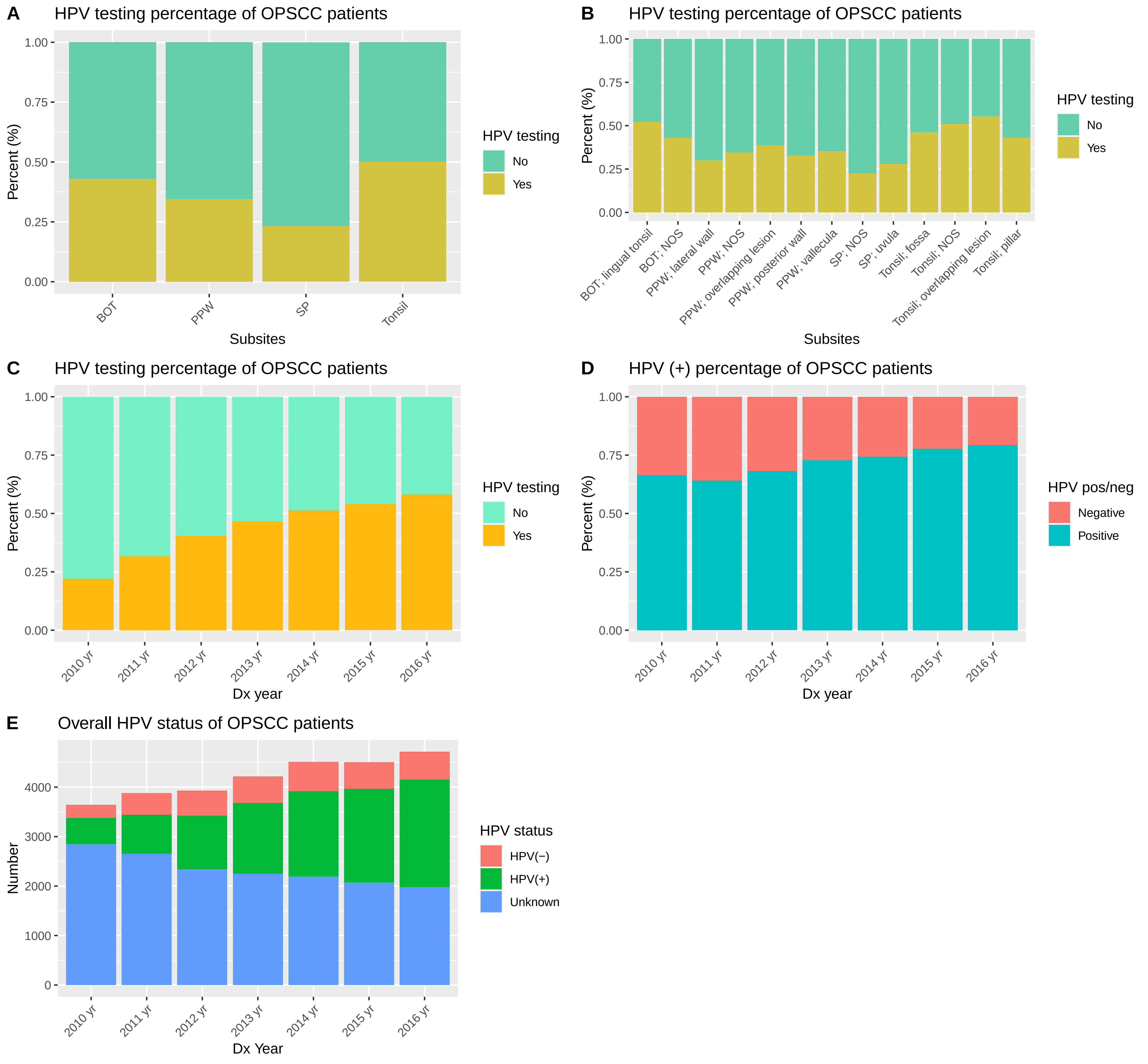

A
HPV testing percentage of OPSCC patients
Percent (%)
1.00
0.75
0.50
0.25
0.00
BOT
PPW
SP
Tonsil
Subsites
HPV testing
No
Yes
B
HPV testing percentage of OPSCC patients
Percent (%)
BOT; lingual tonsil
BOT; NOS
PPW; lateral wall
PPW; NOS
PPW; overlapping lesion
PPW; posterior wall
PPW; vallecula
SP; NOS
SP; uvula
Tonsil; fossa
Tonsil; NOS
Tonsil; overlapping lesion
Tonsil; pillar
Subsites
HPV testing
No
Yes
C
HPV testing percentage of OPSCC patients
Percent (%)
2010 yr
2011 yr
2012 yr
2013 yr
2014 yr
2015 yr
2016 yr
Dx year
HPV testing
No
Yes
D
HPV (+) percentage of OPSCC patients
Percent (%)
Dx year
HPV pos/neg
Negative
Positive
E
Overall HPV status of OPSCC patients
Number
4000
3000
2000
1000
0
Dx Year
HPV status
HPV(−)
HPV(+)
Unknown
