# Supplementary material for: A SEER-based analysis of trends in HPV-associated oropharyngeal squamous cell carcinoma
Source: Infect Agent Cancer. 2024 Jun 28;19:29. doi: 10.1186/s13027-024-00592-5 (PMC11214209; doi:10.1186/s13027-024-00592-5)
Supplement: Supplementary file 4 — Supplementary Material 4: Figure 3. The trends in HPV-positive and HPV-negative patients with OPSCC according to age and primary sites. (a–b) The trends of HPV-positive and HPV-negative patients with OPSCC that originated in BOT, classified according to age group. (c–d) The trends of HPV-positive and HPV-negative patients with OPSCC that originated in PPW, classified according to age group. (e–f) The trends of HPV-positive and HPV-negative patients with OPSCC that originated in SP, classified according to age group. (g–h) The trends of HPV-positive and HPV-negative patients with OPSCC that originated in tonsils, classified according to age group. OPSCC, oropharyngeal squamous cell carcinoma; BOT, base of tongue; PPW, posterior pharyngeal wall; SP, soft palate. [file 13027_2024_592_MOESM4_ESM.pdf]

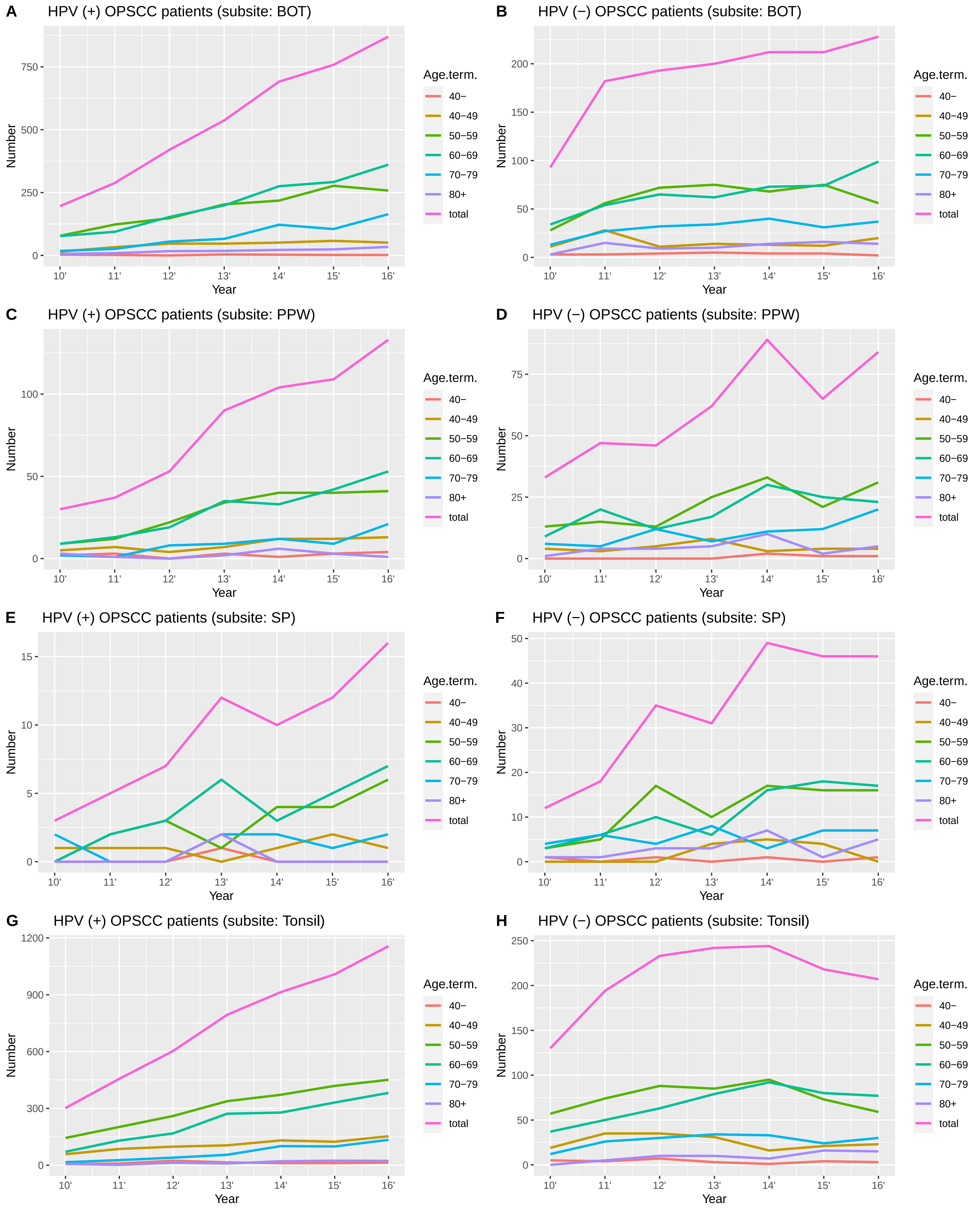
A
HPV (+) OPSCC patients (subsite: BOT)
B
HPV (−) OPSCC patients (subsite: BOT)
C
HPV (+) OPSCC patients (subsite: PPW)
D
HPV (−) OPSCC patients (subsite: PPW)
E
HPV (+) OPSCC patients (subsite: SP)
F
HPV (−) OPSCC patients (subsite: SP)
G
HPV (+) OPSCC patients (subsite: Tonsil)
H
HPV (−) OPSCC patients (subsite: Tonsil)
Number
Year
10'
11'
12'
13'
14'
15'
16'
Age.term.
40−
40−49
50−59
60−69
70−79
80+
total
